# Efficacy and safety of multiple fluocinolone acetonide implants in diabetic macular oedema: comparison between first and second intravitreal injections

**DOI:** 10.1038/s41433-025-03929-5

**Published:** 2025-07-21

**Authors:** Yasmine Serrar, Lucas Sejournet, Victor Thomeret, Corinne Dot, Sophie Bonnin, Vincent Soler, Laurence Rosier, Frédéric Matonti, Mariam Ghazaryan, Thibaud Mathis, Laurent Kodjikian

**Affiliations:** 1 Hôpital de la Croix-Rousse, Hospices Civils de Lyon, Lyon, France; 2 UMR-CNRS 5510 Matéis, Université Lyon 1, Villeurbanne, France; 3 Hôpital Militaire Desgenettes, Lyon, France; 4 Hôpital Édouard-Herriot, Hospices Civils de Lyon, Lyon, France; 5 Académie Militaire du Val de Grâce, Paris, France; 6 Fondation Adolphe de Rothschild, Paris, France; 7 Centre Hospitalier Universitaire de Toulouse, Toulouse, France; 8 Centre Ophtalmologique Rétine Galien, Bordeaux, France; 9 Centre Monticelli Paradis, Marseille, France; 10 Clinique Juge, Groupe Almaviva, Marseille, France; 11 Aix Marseille Univ, CNRS, INT, Inst Neurosci Timone, Marseille, France; 12 Teona Ophthalmology Clinic, Yerevan, Armenia

**Keywords:** Retinal diseases, Metabolic disorders

## Abstract

**Purpose:**

To compare the efficacy and safety between a first and a second injection of fluocinolone acetonide implants (FAc-I) in the treatment of diabetic macular oedema (DMO).

**Methods:**

This retrospective, multicentre cohort study included eyes with chronic DMO that received two consecutive FAc-I injections. We analysed visual and anatomical outcomes, additional DMO treatments, and intraocular pressure (IOP)-related adverse events.

**Results:**

We included 61 eyes from 44 patients. Stable or improved BCVA was observed in 100% of eyes after the first injection and in 93% after the second, demonstrating statistical equivalence within a ±15% margin (*p* = 0.005). Statistical equivalence was also found for the lowest CRT (±10% margin, *p* < 0.001) and for the proportion of eyes with ≥20% CRT decrease (±20% margin, *p* = 0.026). The proportion of patients requiring additional treatments during the first year was equivalent between injections (*p* = 0.036), with a therapeutic burden reduction of 63% and 59%, respectively. Regarding peak IOP, equivalence was observed between the two injections (±5 mmHg margin, *p* < 0.001). The incidence of OHT was numerically higher after the second injection (19.7% vs 11.5%, *p* = 0.302), but this difference was not statistically significant.

**Conclusion:**

Multiple FAc-I injections are a safe and effective treatment option for chronic DMO in real life. The second injection maintained similar functional and anatomical outcomes to the first, supporting the sustainability and repeatability of the treatment.

## Introduction

The prevalence of diabetes is currently increasing worldwide and should reach 12.2% by 2045 [1]. (DMO) is a leading cause of severe visual impairment in the working age population in developed countries [2]. Besides a tight glycaemic and blood pressure control, standard treatment guidelines for DMO include anti-Vascular Endothelial Growth Factor (anti-VEGF) intravitreal injections, intravitreal steroids and laser treatment in specific cases where capillary macroaneurysms are present [3]. Anti-VEGF treatment has been the gold standard treatment for years but it comes with a heavy therapeutic burden as it requires a loading dose of 3–5 monthly injections followed by monthly monitoring visits. Besides, more than 50% of patients have an insufficient response to anti-VEGF treatment, and it has been shown that an early switch from anti-VEGF to DEX-I in these patients is highly beneficial [4, 5].

Intravitreal corticosteroids such as dexamethasone implants (DEX-I) or fluocinolone acetonide implants (FAc-I) reduce levels of VEGF-A but also inhibit several other inflammatory mediators which have an important role in the pathophysiology of DMO, such as IL-6, IL-8, MCP-1, ICAM-1 and TNF-alpha [6]. FAc-I (Iluvien®, Alimera, Nice, France) is a nonbiodegradable tube (3.5 × 0.37 mm) loaded with fluocinolone acetonide (FAc). It is inserted into the vitreous with a 25-gauge needle and it delivers 0.2 µg of FAc per day. FAc-I has been approved for the treatment of DMO in patients who have previously received conventional treatment without sufficient response, and it aims to reduce not only treatment burden but also anatomical fluctuations as it delivers stable doses of the drug in the vitreous up to 36 months [7, 8].

Efficacy and tolerance of FAc-I have been evaluated in the FAME clinical trial, and in real-world studies [8–10]. Some studies evaluated the long-term efficacy and safety of FAc-I, showing sustained BCVA gains over 3 years after FAc-I and anatomical improvement up to 5 years [11]. However, in the literature, there is no data on eyes treated with multiple injections of FAc-I. Our aim was to evaluate and compare the efficacy and tolerance of FAc-I after a first and a second injection to determine whether multiple FAc-I injections may lead to a repeatable and sustainable response, and thus to stable anatomical and functional outcomes.

## Materials and methods

Our study is a retrospective, multicentre, French study. Patients were recruited between July 2018 and January 2024 in several retinal reference centres across France (Hospices Civils de Lyon; Fondation Rothschild-Paris; CHU Toulouse; Rétine Gallien-Bordeaux; Centre Monticelli-Marseille; CHU Bordeaux; CHU Nantes; CHU Grenoble; CHU Nancy; CHU Dijon; CHI Créteil; CHU Amiens; CH Niort). We included eyes of patients that received at least two consecutive FAc-I for chronic DMO with an insufficient response to previous treatments, in accordance with the European marketing authorisation. Informed consent was obtained from all subjects involved in the study. An international review board approved this study (Ethics Committee of the French Society of Ophthalmology, IRB 00008855 Société Française d’Ophtalmologie IRB#1).

### Study population

We included consecutive patients with a history of chronic macular oedema secondary to type 1 or type 2 diabetes, over 18 years old, who received two consecutive FAc-I injections in the same eye. Eyes with other causes of macular oedema than DMO (retinal vein occlusion, uveitis, pseudophakic macular oedema, radiation maculopathy) were not eligible. Both eyes of the same patient were included if they met all the inclusion criteria.

### Treatment regimen

FAc-I injections were administered either alone or in combination with DEX-I at the clinician’s discretion. When a DEX-I injection was combined, it was performed on the same day as the FAc-I injection or during the following month. The interval between the two FAc-I injections was decided by the ophthalmologist based on the patient’s clinical assessment. During the follow-up, additional treatments could be prescribed (DEX-I intravitreal injection, anti-VEGF intravitreal injection or focal macular laser treatment). The management of ocular hypertension (OHT) was based on the clinician’s judgement.

### Data collection

Data were collected retrospectively by looking into the medical records. Collected information at baseline included: age, gender, general medical history, ophthalmological history, prior DMO treatments. The following data was collected at every follow-up visit: best corrected visual acuity (BCVA) using Early Treatment Diabetic Retinopathy Study (ETDRS) charts, intraocular pressure (IOP), Central subfield Retinal Thickness (CRT) measured in the central 1 mm-circle on macular Optical Coherence Tomography, additional treatments, IOP-lowering medications, as well as any declared side effect or adverse event.

### Outcomes

For each eye, we compared the first and the second FAc-I injection based on several outcomes. The main outcome was the proportion of eyes with a stable or improved BCVA; improved BCVA was defined as a gain of at least one letter, and stable BCVA as a loss of no more than five letters between pre-injection BCVA and the maximum BCVA obtained during follow-up. Secondary outcomes included the BCVA during follow-up, the lowest CRT reached during follow-up, and the proportion of eyes obtaining a CRT decrease of at least 20% compared to baseline. We also analysed the proportion of eyes requiring any additional treatment during follow-up, the mean number of additional treatments received in the 12 months following each FAc-I injection, and the cumulative risk of additional treatment, in the same period. To evaluate the tolerance of FAc-I in multi-injected eyes, we compared the maximum IOP reached after each FAc-I injection.

### Statistical analysis

No a priori sample size calculation was performed for this retrospective real-world study. However, a post hoc power analysis showed that for the primary endpoint (stable/improved BCVA: 100% vs. 93%), the sample of 61 eyes provided 82.7% power to demonstrate equivalence within a ±15% margin at *α* = 0.05.

Quantitative variables were expressed as mean ± standard deviation (SD), unless otherwise specified, and qualitative variables as counts (percentages). Comparisons between groups were performed using the Wilcoxon signed-rank test for quantitative variables and the McNemar test for qualitative variables. The cumulative risk of requiring additional treatments during follow-up was analysed using a time-to-event model, and comparisons were tested with a log-rank test.

The primary objective was to assess the equivalence between two FAc-I injections across functional, anatomical, and treatment-related criteria. *P*-values were corrected within each family of criteria using the Dirak method to account for multiple comparisons. All statistical tests were two-sided, including equivalence tests based on the TOST procedure. The null hypothesis of no difference (NHST) and the two one-sided tests (TOST) were performed at *α* = 0.05 significance level.

For the primary outcome, one test was performed with the following equivalence margin between injections: ±15% for BCVA stability or improvement. For secondary functional outcomes, one test was performed with an equivalence margin of ±5 letters for BCVA. For secondary anatomical outcomes, two tests were performed: equivalence was tested within a ±10% margin for the reduction in minimum CRT, and a ±20% margin for CRT reduction ≥20%. For secondary treatment-related outcomes, two tests were performed: the equivalence in number of additional treatments was tested within ±1 treatment. For secondary safety outcomes, one test was performed with an equivalence margin of ±5 mmHg for highest IOP. The incidence of steroid-induced OHT after the first and second FAc-I injections was compared using McNemar’s test for paired proportions.

All figures are based on appropriate statistical tests. For paired continuous variables, the Wilcoxon signed-rank test was used after checking for non-normality (Shapiro–Wilk test, QQ-plots); for paired binary outcomes, exact McNemar tests and TOST for equivalence were used. The variation within each group is reported as mean ± standard deviation, and displayed in tables and figure error bars (defined as SD). Homogeneity of variance was not assumed due to the use of nonparametric tests.

## Results

### Population characteristics

We included a total of 61 eyes of 44 patients from 14 centres in France, treated with two consecutive FAc-I between December 2018 and January 2024. At baseline, the mean duration of DMO was 63.4 ± 37.0 months. (Table 1**)** All eyes had previously received at least one DEX-I and the mean number of DEX-I before the first FAc-I injection was 7.4 ± 4.5. Almost all eyes (93.4%, *n* = 57) were pseudophakic. Eleven eyes (18.0%) had glaucoma or OHT, and two eyes (3.3%) had a history of glaucoma surgery (one trabeculectomy and one mini-invasive glaucoma surgery). Seventeen patients (27.9%) received bilateral FAc-I injection, whether simultaneously or not.

**Table 1:** Patients’ characteristics at baseline.

Age, years (mean ± SD)	68.5 ± 8.7
Male, *n* (%)	29 (47.5)
Type of diabetes, *n* (%)	
type 1	8 (13.1)
type 2	53 (86.9)
Diabetes duration, years (mean ± SD)	22.5 ± 12.5
Insulin therapy (pump/injection), *n* (%)	40 (66.7)
HbA1c, % (mean ± SD)	7.6 ± 1.1
High Blood Pressure, *n* (%)	46 (75.4)
Obstructive sleep apnoea, *n* (%)	7 (11.5)
DMO duration, months (mean ± SD)	63.4 ± 37.0
Diabetic Retinopathy stage, *n* (%)	
mild NPDR	1 (1.6)
moderate NPDR	12 (19.7)
severe NPDR	8 (13.1)
PDR	2 (3.3)
complete PRP	38 (62.3)
PRP (partial or complete) *n* (%)	47 (77.0)
Pseudophakic, *n* (%)	57 (93.4)
History of vitrectomy, *n* (%)	16 (26.2)
Epiretinal membrane, *n* (%)	31 (50.8)
No surgery	25 (41.0)
Performed surgery	6 (9.8)
Glaucoma and/or OHT, *n* (%)	14 (23.0)
Baseline IOP, mmHg (mean ± SD)	14.0 ± 3.1
IOP-lowering treatments, *n* (%)	
Topical medication	10 (16.4)
History of SLT	0
History of IOP-lowering surgery	2 (3.3)
Focal macular laser treatment, *n* (%)	16 (26.2)
DEX-I intravitreal injections before FAc-I, *n* (%)	61 (100)
Number of injections (mean ± SD)	7.4 ± 4.5
Interval between injections, months (mean ± SD)	4.5 ± 1.4
Anti-VEGF intravitreal injections before FAc-I, *n* (%)	49 (80.3)
Number of injections (mean ± SD)	9.8 ± 6.8
Ranibizumab, *n* (%)	32 (52.5)
Aflibercept, *n* (%)	33 (54.1)
Bevacizumab, *n* (%)	4 (6.6)

*DEX-I* intravitreal dexamethasone implant, *DMO* diabetic macular oedema, *FAc-I* intravitreal fluocinolone acetonide implant, *HbA1c* glycated haemoglobin, *IOP* intraocular pressure, *NPDR* non proliferative diabetic retinopathy, *OHT* ocular hypertension, *PDR* proliferative diabetic retinopathy, *PRP* panretinal photocoagulation, *SLT* selective laser trabeculoplasty, *VEGF* vascular endothelial growth factor.

The mean interval between the first and second FAc-I injections was 25.7 ± 7.7 months. The mean follow-up was 23.8 ± 6.8 months after the first FAc-I injection and 16.5 ± 7.5 months after the second one. In our cohort, five eyes (8.2%) received a third FAc-I injection (data were not collected after a third injection).

At baseline, the mean BCVA was 61.8 ± 15.5 letters before the first FAc-I injection and 63.2 ± 17.2 letters before the second FAc-I injection. (Table 2) The mean CRT was 409.9 ± 151.3 before the first FAc-I injection and 354.0 ± 105.2 letters before the second FAc-I injection. Concerning IOP, the proportion of patients treated with at least one IOP-lowering medication was 16.4% (*n* = 10) before the first FAc-I injection and 34.4% (*n* = 21) before the second FAc-I injection. The first FAc-I injection was combined with a DEX-I injection in 26.2% of patients (*n* = 16). The second FAc-I injection was combined with a DEX-I injection in 54.1% of patients (*n* = 33).

**Table 2:** Baseline characteristics before first and second FAc-I injections.

	First FAc-I injection	Second FAc-I injection
BCVA before FAc-I injection, letters (mean ± SD)	61.8 ± 15.5	63.2 ± 17.2
Baseline IOP, mmHg (mean ± SD)	14.0 ± 3.1	13.6 ± 3.2
IOP lowering medication, *n* (%)	10 (16.4%)	21 (34.4%)
monotherapy	4 (6.6%)	7 (11.5%)
dual therapy	5 (8.2%)	13 (21.3%)
triple therapy	1 (1.6%)	2 (3.3%)
quadruple therapy	0	0
Baseline CRT, µm (mean ± SD)	409.9 ± 151.3	354.0 ± 105.2
Combined DEX-I injection, *n* (%)	16 (26.2%)	33 (54.1%)

*BCVA* best corrected visual acuity, *DEX-I* intravitreal dexamethasone implant, *FAc-I* intravitreal fluocinolone acetonide implant, *IOP* intraocular pressure, *CRT* Central Retinal Thickness.

### Functional outcomes

**Fig. 1:**
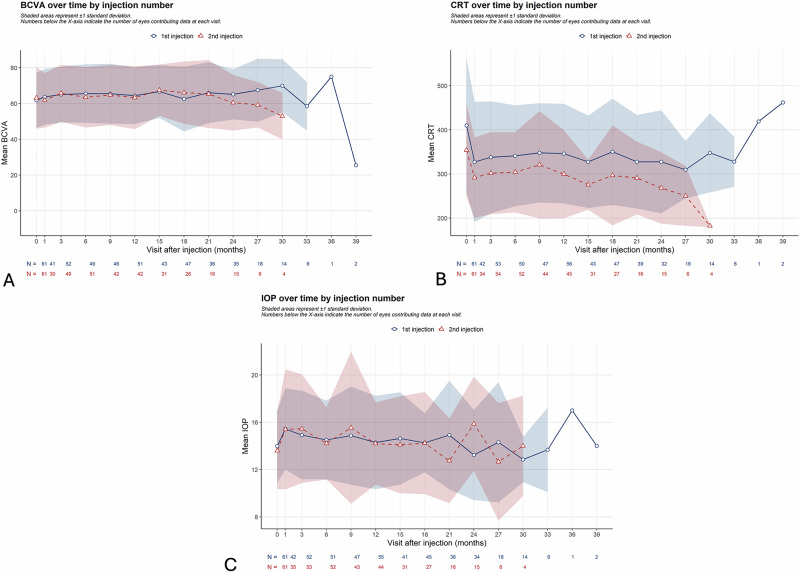
Mean change in best-visual acuity (BCVA), central macular thickness (CMT) and intra-ocular pressure (IOP) after first and second fluocinolone-acetonide implant injections (FAc-I). **A** Mean change in BCVA. **B** Mean change in CMT. **C** Mean change in IOP. BCVA = best corrected visual acuity, CRT central retinal thickness, FAc-I intravitreal fluocinolone acetonide implant, IOP intraocular pressure.

The mean peak BCVA was 71.6 ± 13.6 letters after the first FAc-I injection and 68.2 ± 14.3 letters after the second one. Concerning the primary outcome, 61 (100%) patients had a stable or improved BCVA after the first FAc-I injection and 56 (93%) patients met the same criteria after the second FAc-I injection. This outcome demonstrated statistical equivalence with an equivalence margin of ±15% (*p* = 0.005) (Table 3). The comparison of BCVA after the first and second FAc-I injections also showed equivalence within an equivalence margin of ±5 letters (*p* = 0.016). The mean BCVA reached 65 letters at 3 months after both first and second injections and then formed a plateau, sustained until month 30 (first FAc-I injection) and month 21 (second FAc-I injection) (Fig. 1).

**Table 3:** Comparison of efficacy and safety outcomes after first and second FAc-I injections with equivalence margins.

Outcome	Variables	First FAc-I injection	Second FAc-I injection	Equivalence margin	Crude *p*-value for equivalence	Corrected *p*-value* for equivalence
Primary outcome	BCVA stable or improved, *n* (%)	61 (100%)	56 (93%)	±15%	0.005	0.005
Secondary outcomes	Peak BCVA after FAc-I injection, letters (mean ± SD)	71.6 ± 13.6	68.2 ± 14.3	±5 L	0.008	0.008
	Lowest CRT, µm (mean ± SD)	274.1 ± 68.0	266.6 ± 66.7	±10%	<0.001	<0.001
	CRT decrease >20% *n* (%)	34 (55.7%)	28 (45.9%)	±20%	0.013	0.026
	Number of additional treatments, (mean ± SD)	1.2 (2.0)	1.2 (1.8)	±1 additional treatment	<0.001	<0.001
	Patients requiring additional treatment during follow-up, *n* (%)	27 (45.9%)	28 (44.3%)	±15%	0.018	0.036
	Peak IOP, mmHg (mean ± SD)	18.6 ± 4.9	18.7 ± 5.3	±5 mmHg	<0.001	<0.001

*BCVA* best corrected visual acuity, *CRT* Central Retinal Thickness, *DEX-I* intravitreal dexamethasone implant, *FAc-I* intravitreal fluocinolone acetonide implant, *IOP* intraocular pressure, *OHT* ocular hypertension, *SLT* selective laser trabeculoplasty.

^*^Correction is: $${Corrected\; pvalue}=1-{(1-{Crude\; pvalue})}^{{Number\; of\; tests\; in\; the\; family}}$$.

### Anatomical outcomes

The lowest CRT was 274.1 ± 68.0 µm after the first FAc-I injection and 266.6 ± 66.7 µm after second FAc-I injection. These criteria showed statistical equivalence with a margin of ±10% (*p* < 0.001) (Table 3). The proportion of eyes achieving a CRT reduction of at least 20% compared to baseline was also equivalent after first and second FAc-I injections with a margin of ±20% (*p* = 0.0026). The mean CRT rapidly decreased after both FAc-I injections with a trough at 327.2 µm (first FAc-I injection) and 291.8 µm (second FAc-I injection) and was then followed by a plateau (Fig. 1).

### Additional treatments

To account for the shorter follow-up after the second FAc-I injection, we analysed the additional treatments during the first 12 months after both FAc-I injections. The proportion of patients requiring at least one additional treatment during the first year after the first and second FAc-I injections were statistically equivalent (*p* < 0.001). (Supplemental Fig. 1). The results also showed significant equivalence in the number of additional treatments between the first and second FAc-I injections with a margin of ±1 additional treatment (*p* = 0.036) (Table 3).

The mean interval between two DEX-I injections was estimated at 4.5 ± 1.4 months before any FAc-I injection. After the first FAc-I injection, the mean interval between two DEX-I injections increased to 12.5 ± 9.6 months, corresponding to a 63% reduction of the therapeutic burden. After the second FAc-I injection, the mean interval between two DEX-I injection was 11.3 ± 8.0 months, corresponding to a 59% reduction of the therapeutic burden. When considering all type of additional treatments, the interval between any additional treatment was 11.2 ± 9.9 months after the first FAc-I injection and 9.8 ± 8.1 months after the second FAc-I injection.

### IOP outcomes

Regarding the peak IOP after FAc-I, equivalence was found between the first and second FAc-I injections with a margin of ±5 mmHg (*p* < 0,001). (Table 3). The proportion of steroid-induced OHT (defined as an IOP ≥ 25 mmHg and/or an IOP increase ≥10 mmHg) was 11.5% (*n* = 7) after the first FAc-I injection, and 19.7% (*n* = 12) after the second one (*p* = 0.302). Among the OHT after FAc-I, OHT occurred in the 3 months following a DEX-I injection in thre patients (*n* = 3/7, 43%) after the first FAc-I injection, and in six patients (*n* = 6/15, 40%) after the second FAc-I injection.

**Fig. 2:**
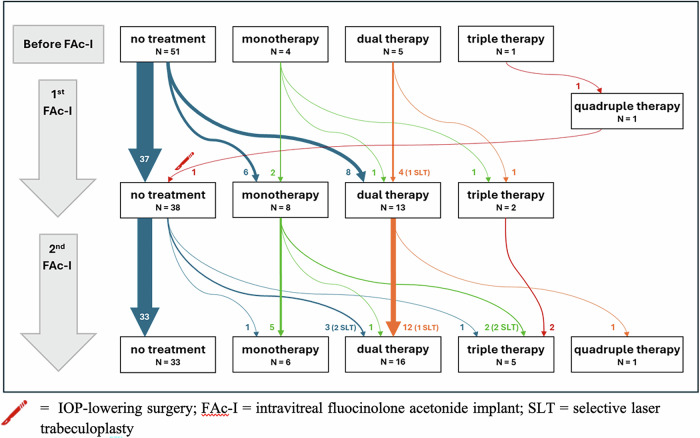
Management of IOP-lowering treatment after first and second FAc-I injections.

Most cases of OHT after FAc-I injection were treated either with local hypotensive medication or selective laser trabeculoplasty (SLT). There was only one IOP-lowering surgery reported in our study, after the first FAc-I injection (Fig. 2). The mean IOP peaked at 15.4 mmHg a month after each FAc-I administration and remained stable afterwards (Fig. 1).

### Other safety outcomes

Out of the four eyes that were phakic at baseline, all of them underwent cataract surgery after the first FAc-I injection, at a mean time of 9.0 ± 1.4 months.

No endophthalmitis was reported after the first FAc-I injection. One case occurred after the second one, 6.3 months after the FAc-I injection and 1.2 months after a DEX-I injection.

## Discussion

The present study is the first one to compare the efficacy and tolerance of the first and second FAc-I injections in eyes treated with multiple FAc-I injections for chronic DMO. The main goal was to assess whether the second injection was as efficient and as well tolerated as the first one. This cohort of 61 eyes treated with multiple FAc-I injections is large considering the fact that the device has been used in France since 2019 only, and that it is supposed to be effective for about 3 years. Patients’ demographics and ocular history were similar to those observed in the FAME trial [12]. The main difference from the FAME study population is that glaucoma, OHT or concurrent IOP-lowering drops were exclusion criteria in the pivotal trial. Our population was characterised by a longer-standing DMO (3.6 years in FAME versus 5.3 years in our study). Other differences include the proportion of phakic patients (65.2% in FAME versus 6.6% in our study) and the mean BCVA at baseline (53.4 letters in FAME versus 61.8 letters in our study). Our higher baseline BCVA can be explained by the fact that most patients in our cohort were already well treated with DEX-I before FAc-I injection, contrary to FAME patients who were probably having oedema recurrence at baseline [10]. This also explains why BCVA gains measured in our study were not as high as those in FAME. When compared with other large-scale real-world studies, our population had very comparable clinical characteristics [13–15].

We showed that the proportion of patients with a stable or improved BCVA was similar after the first and the second FAc-I injections. Regarding the anatomical outcomes, we found statistical equivalence with a margin of ±10% in the lowest CRT obtained after the first and second injections, with figs approaching those measured in healthy eyes [16]. The proportion of eyes achieving a CRT reduction of at least 20% (accepted threshold for defining an anatomical non-responder) were equivalent with a margin of ±20% after the two injections either [17]. In our study, the mean BCVA and CRT showed an improvement between baseline and month 3 followed by a long-standing plateau, and the same pattern was observed after the second injection. Stabilising patients is the main purpose when treating DMO with FAc-I. Due to the lipophilic nature of the molecule and its slow-release into the vitreous, continuous low doses of FAc are delivered to the retina up to 36 months after injection [7]. During that period, FAc-I allows to reduce both anatomical and visual fluctuations [6]. This is particularly important as CRT fluctuations may worsen final BCVA [18, 19]. Indeed, it was shown that anatomical recurrence of DMO precedes functional recurrence by a mean of 17.5 days in a prospective study about DEX-I [20]. Consequently, a proactive DEX-I injection regimen, where DEX-I was injected just before the anatomic recurrence, showed significant improvement of functional and anatomical outcomes compared to the usual Pro Re Nata regimen [21]. This aligns with our study that shows that two FAc-I injections help stabilise both anatomical and functional outcomes in patients with chronic DMO. However, the mean peak BCVA was slightly lower after the second FAc-I injection (68.2 vs 71.6 letters) despite meeting predefined equivalence criteria. This may be explained by the minor CRT fluctuations observed at the beginning and end of the follow-up period, as shown in Fig. 1. As previously mentioned, anatomical fluctuations are known to be associated with poorer visual outcomes [18, 19]. This finding further emphasises the need for tight anatomical control in the long-term management of DMO.

DMO treatment usually comes with a heavy therapeutic burden, and reducing that burden is another major goal achieved after FAc-I implant according to most studies [6, 22]. Guidelines on FAc-I in DMO written by an international experts’ panel were recently published and establish FAc-I as a background therapy when DEX-I should be used as an ‘attack’ therapy, using an analogy with migraine management [23]. Our results show lower therapeutic burden after FAc-I, with a reduction by 63% of the number of DEX-I injections after the first FAc-I injection, and by 59% after the second one. Additionally, 55.7% of the studied eyes after the first FAc-I injection and 54.1% after the second FAc-I injection remained free of any additional treatment at month 12. These results show multiple FAc-I injections may help reduce the therapeutic burden and help provide a better quality of life for patients with chronic DMO [24]. This is of particular importance for diabetic patients, who tend to have numerous follow-up visits with a large variety of healthcare professionals, and a lower compliance compared to patients treated with intravitreal injections for other causes [25, 26].

The safety of FAc-I regarding IOP was an important concern in our study, as we included 18% of eyes with a history of OHT and/or glaucoma. It was shown that the absence of OHT after DEX-I injections has a very good predictive value for the absence of OHT after FAc-I [14, 27]. It is usually recommended to test the corticosteroid-induced IOP response with two to three DEX-I injections prior to FAc-I injection [28]. All patients in our cohort received at least one DEX-I before FAc-I injection, with a mean of 7.4 DEX-I injections. Thus, patients had already been screened for steroid-induced OHT, and most high responders probably were contra-indicated to FAc-I. This is why the OHT rates in our study (11.5% after the first FAc-I injection and 24/6% after the second FAc-I injection) were lower than those observed in the FAME trial [12]. Most cases of OHT after FAc-I injection were managed with SLT or topical hypotensive medication according to the guidelines for the management of OHT induced by intravitreal injections of corticosteroids that were issued by the French Society of Ophthalmology in 2023 [28]. In our study, only one case of IOP-lowering surgery was reported (trabeculectomy). It occurred one year after the first FAc-I injection. This patient had a 10-year history of glaucoma, was under triple therapy at baseline, and was known to be a high IOP-responder to DEX-I. He was treated with FAc-I despite these risk factors for OHT as he presented a severe DMO with no response to anti-VEGF intravitreal injections. After the surgery, the patient had a good IOP control which allowed a second FAc-I injection without recurrence of OHT during follow-up. It has been shown intravitreal corticosteroids may be used on eyes with a history of filtering surgery in complex situations with no effective alternative to treat macular oedema [29].

Our study showed a numerically higher incidence of OHT following the second FAc-I injection compared to the first (19.7% vs 11.5%), although the difference was not statistically significant (*p* = 0.302). This trend may be related to the greater proportion of patients who received DEX-I in combination with the second FAc-I compared to the first FAc-I injection (54.1% vs 26.2%). We also observed that 43% of OHT after the first FAc-I injection, and 40% of OHT after the second FAc-I injection, occurred in the 3 months following a DEX-I injection (whether it be an additional treatment or a DEX-I implant combined with a FAc-I injection). This temporal association is consistent with findings from the MEAD study, where the greatest increase in IOP following a DEX-I implant occurred within 3 months, before returning to baseline by 6 months [30]. The risk of OHT may therefore be increased when FAc-I injections are repeated, particularly in combination with or shortly after DEX-I. Taken together, these findings support a potential cumulative or time-dependent effect of DEX-I on OHT, especially within the first three months after exposure. This also echoes the results of the REALFAC study that suggested a cumulative effect of steroids on the incidence of OHT [10]. A retrospective study by Billant et al. showed a prevalence of late steroid-induced OHT of 20.8% among a cohort of patients treated with multiple DEX-I injections. Sixty-five percent of OHT spikes occurred between the fourth and sixth intravitreal injection and 35% occurred later. This shows that even if steroid-induced OHT is a manageable side effect of DEX-I or FAc-I injections, it is important to ensure regular follow-up to check for the absence of late-onset OHT [31].

This study has several limitations. The first limitation is the short follow-up after the second FAc-I injection. Indeed FAc-I is a relatively recent drug on the market, especially in France. That may have created bias regarding certain outcomes, as the second implant may not have reached its full efficiency in the shorter follow-up period. We tried to reduce that shorter follow-up limitation whenever possible. This is why we analysed additional treatments in the first 12 months only, after each FAc-I injection, in order to compare the same duration each time. The second limitation of our study it due to its retrospective nature: a few data were missing during collection. Some visits may have been skipped, and some information may have missed at certain visits. Then, it is likely that the use of DEX-I as an additional treatment had an impact on our results. Indeed, our clinicians followed the same approach as the one outlined in the international recommendations mentioned above [23]. As a result, we did not evaluate FAc-I as a monotherapy, but in combination with DEX-I. Subgroup analyses restricted to DEX-I–free patients or stratified by treatment burden were not feasible due to limited sample size and absence of clearly defined subgroups. However, our aim was to assess the use of FAc-I in real-life practice, where it is commonly used alongside additional therapies including DEX-I. Moreover, OHT after FAc-I could partly be underestimated, as we were not able to retrospectively identify the number of patients who were excluded from receiving a second FAc-I injection due to prior severe OHT. These patients likely represent a population at higher risk, which was not included in the second-injection cohort. This limitation has been acknowledged and may have influenced the OHT rates reported. Finally, patients’ management including follow-up scheme, management of additional treatments and IOP-lowering medication, was left at the physician’s discretion and could differ among the centres. However, all the patients in our cohort were treated and followed in reference tertiary centres, by retina specialists, who were most likely well aware of the most recent guidelines and follow-up standards established by pivotal trials [8, 23, 28].

## Conclusion

This retrospective, observational cohort study is the first one to focus specifically on multiple intravitreal injections of FAc-I in patients with chronic DMO in real-life. It supports the efficacy, safety, sustainability and repeatability of FAc-I injections, granting long-term visual and anatomical stabilisation, with low safety concerns. We also found that multiple FAc-I injections help reduce the therapeutic burden for patients with chronic DMO. These results are comparable with previous data from other real-world studies and need to be confirmed with a longer follow-up in eyes treated with two FAc-I injections or more.

Supplemental material is available at Eye’ website.

## Summary

### What was known before:

- In this study, we evaluated the long-term efficacy and safety of multiple intravitreal injections of the fluocinolone acetonide implant (FAc-I) in patients with (DMO).
- Our retrospective, multicentre cohort included 61 eyes from 44 patients who received two consecutive FAc-I injections.
- We compared both functional and anatomical outcomes between the first and second injections to assess their equivalence.

### What this study adds:

- To our knowledge, no equivalent study has yet been published as there is no data on eyes treated with multiple injections of FAc-I.
- The present study is the first one to compare the efficacy and tolerance of the first and second FAc-I injections in eyes treated with multiple FAc-I injections for chronic DME.
- Moreover, in the present study, the number of patients for this type of long-acting drug is quite high, especially considering the fact that the device has been used in France since 2019 only and that it is supposed to be effective for about 3 years.

## Supplementary information

Supplemental Fig 1: Additional treatments received in the 12 months following the first and second FAc-I injections.

## Data Availability

The datasets generated during and/or analysed during the current study are available from the corresponding author on reasonable request.
