# Supplementary material for: Efficacy and safety of multiple fluocinolone acetonide implants in diabetic macular oedema: comparison between first and second intravitreal injections
Source: Eye (Lond). 2025 Jul 21;39(14):2678–85. doi: 10.1038/s41433-025-03929-5 (PMC12446434; doi:10.1038/s41433-025-03929-5)
Supplement: Supplementary file 1 — Supplemental Fig 1: Additional treatments received in the 12 months following the first and second FAc-I injections. [file 41433_2025_3929_MOESM1_ESM.docx]

**Supplemental Material**

**Supplemental Figure 1: Additional treatments received in the 12 months following the first and second FAc-I injections**

**
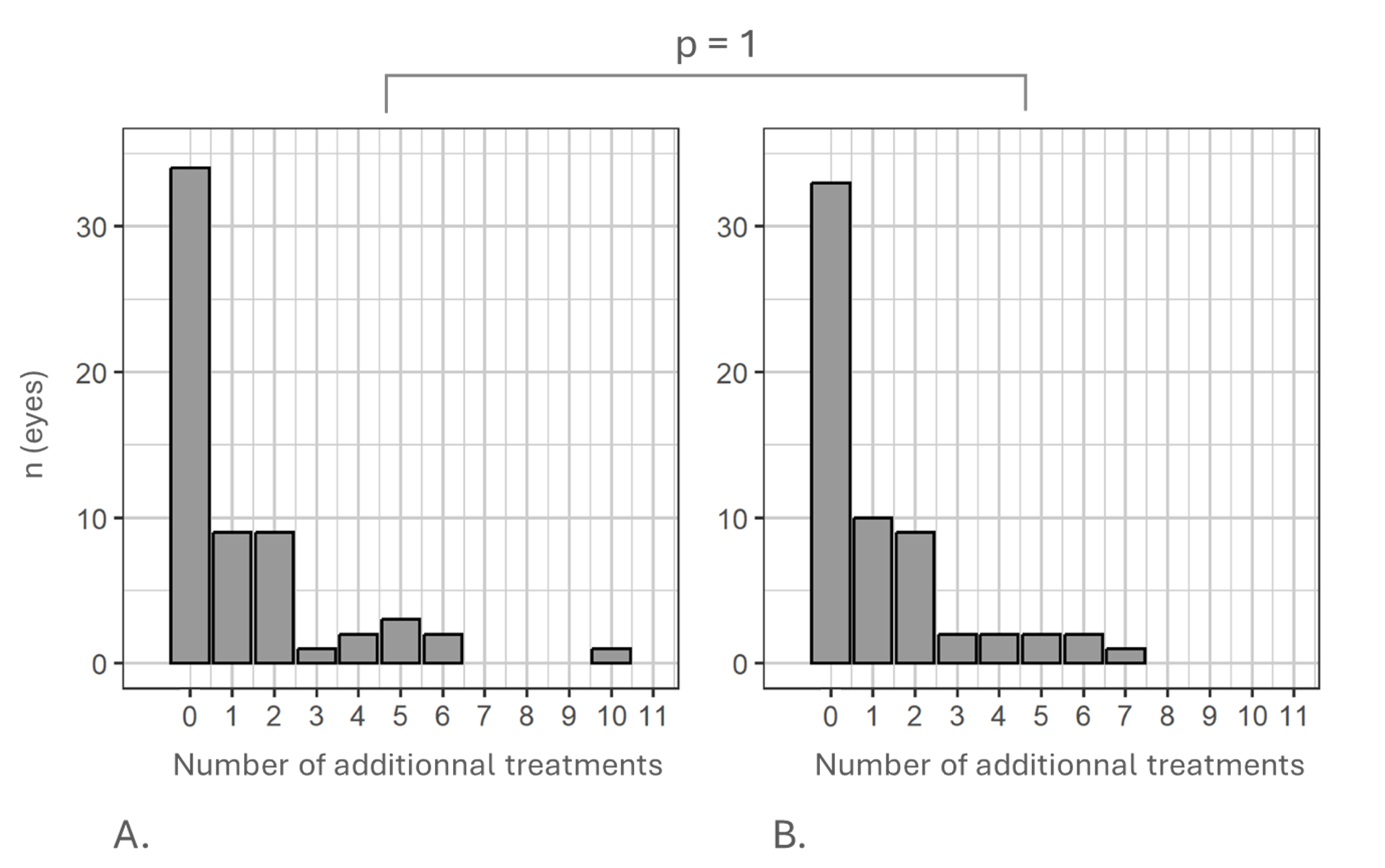
**

1. Additional treatments distribution after the first FAc-I injection
2. Additional treatments distribution after the second FAc-I injection
